# How Poor Is Your Sample? A Simple Approach for Estimating the Relative Economic Status of Small and Nonrepresentative Samples

**DOI:** 10.9745/GHSP-D-22-00394

**Published:** 2023-04-28

**Authors:** Jan Ostermann, Nicole Hair, Volker Grzimek, Siyu Zheng, Wenfeng Gong, Kathryn Whetten, Nathan Thielman

**Affiliations:** a University of South Carolina, Columbia, SC, USA.; b Berea College, Berea, KY, USA.; c Johns Hopkins University, Baltimore, MD, USA.; d Duke University, Durham, NC, USA.

## Abstract

**Background:**

Asset-based indices of living standards, or wealth indices, are widely used proxies for economic status; however, such indices are not readily available for small and nonrepresentative samples.

**Methods:**

We describe a simple out-of-sample prediction approach that uses estimates from large and representative “reference” samples to calculate measures of relative economic status (e.g., wealth index scores) for small and/or nonrepresentative “target” samples. The method relies on the availability of common variables and assumptions about comparable associations between these variables and the underlying construct of interest (e.g., household wealth). We provide 2 sample applications that use Demographic and Health Surveys (DHS) from 5 countries as reference samples. Using ordinary least squares regression, we estimate associations between household characteristics and the DHS wealth index. We use parameter estimates to predict wealth index scores for small nonrepresentative target samples. Comparisons of wealth distributions in the reference and target samples highlight selection effects.

**Results:**

Applications of the approach to diverse populations, including populations at high risk of HIV infection and households with orphaned and separated children, demonstrate its usefulness for characterizing the economic status of small and nonrepresentative samples relative to existing reference samples. Women and men in northern Tanzania at high risk of HIV infection were concentrated in the upper half of the wealth distribution. By contrast, the relative distribution of household wealth among households with orphaned and separated children varied greatly across countries and rural versus urban settings.

**Conclusions:**

Public health professionals who implement, manage, and evaluate programs in low- and middle-income countries may find this approach applicable because of the simplicity of the estimation methods, low marginal cost of primary data acquisition, and availability of established measures of relative economic status in many publicly available household surveys (e.g., those administered by the DHS Program, World Bank, International Labour Organization, and UNICEF).

## Abstract (precis)

The authors demonstrate the simplicity and utility of a method for estimating the relative economic status of small and nonrepresentative samples relative to existing representative reference populations.

## Teaser

**Key Findings:**

- Survey data from small and nonrepresentative samples often do not allow for robust inferences about respondents’ relative economic well-being.
- We demonstrate that out-of-sample predictions based on a straightforward analysis of publicly available data and a small number of purposefully selected survey questions provide robust estimates of relative household wealth for small and nonrepresentative samples.
- The approach minimizes uncertainty over how to weigh diverse household characteristics and concerns about the precision and external comparability of estimates derived from small and nonrepresentative samples.

**Key Implications:**

- Program managers should consider this method for describing inclusion and exclusion errors for social assistance programs.
- Program evaluators could use the method to describe sampling and selection biases in small target populations.

## BACKGROUND

Asset-based indices of living standards, also referred to as wealth indices,^1^ are frequently used proxy measures of socioeconomic status, particularly in low- and middle-income countries (LMICs). Wealth indices use a series of assets and other household characteristics to describe a latent construct (e.g., economic status) or an unmeasured variable (e.g., household wealth).

Filmer and Scott, and more recently Poirier et al., described the widespread use of asset-based indices to characterize associations between households’ economic status and diverse education, health, labor market, and other outcomes.^2^^,^^3^ Wealth index scores are commonly derived using principal components analysis on a range of household assets and other characteristics, although other methods have been used, such as factor analysis or asset counts.^4^^–^^6^ To date, wealth index scores have been used primarily in large and often nationally representative data sets.^7^^–^^20^ Wealth indices are rarely constructed for small and nonrepresentative samples because within-sample estimates of the contributions of diverse characteristics to household wealth cannot be validated, and the estimated wealth index scores in the sample cannot be compared to the distribution of wealth in the general population. Recognizing that program implementers and evaluators often have difficulty collecting and analyzing data on program beneficiaries’ wealth, recent methodology reports have described the construction of simplified asset indices that adapt the well-known Demographic and Health Surveys (DHS) wealth index.^21^^–^^23^ These reports show that a simplified asset index (based on a much-reduced version of the DHS asset questionnaire) can provide a good approximation to the DHS wealth index and wealth quintiles while being much easier to administer. These strategies require complete information about how the wealth index and cut-off points were constructed in a particular DHS program survey^22^ or the prospective adoption of an automated tool.^21^^,^^24^

In this article, we present an alternative approach: we demonstrate that a simple out-of-sample prediction approach can be used to estimate externally comparable measures of relative economic status for small and nonrepresentative samples. Like simplified asset indices, this approach relies on the availability of component weights from a “reference” population and an assumption that associations between the components of the index (e.g., assets) and the construct it intends to measure (e.g., household wealth or economic status) are the same in the reference and “target” populations. This approach has several advantages. First, it does not require knowledge of the details about the construction of the reference index, and yet, predictions will be on the same scale as the reference index, and the relative status of households or individuals can be directly compared between the reference and target samples. Second, it can be applied both in countries with a recent DHS survey and in countries with any other nationally or regionally representative data source that includes information about household characteristics and economic position. Third, in cases where the contributions of specific household assets to economic well-being change over time, it can be applied retrospectively to historical data and adapted as new data become available. With the adoption of a harmonized reference index,^25^^–^^27^ it is amenable to intertemporal and/or cross-national comparisons. Finally, the approach is not constrained to measures of household wealth, as it could be similarly applied to other measures of relative economic status derived from income, consumption, or expenditure data.

We demonstrate the use of a simple out-of-sample prediction approach to estimate externally comparable measures of relative economic status for small and nonrepresentative samples.

In 2 sample applications, we demonstrate the utility and simplicity of the method, using the DHS as reference data. Specifically, we estimate the contributions of household assets and other household characteristics to the DHS wealth index and use the estimated parameters to make out-of-sample predictions of wealth index scores for small nonrepresentative survey samples from Cambodia, Ethiopia, India, Kenya, and Tanzania.^28^^,^^29^ This approach may be a useful tool for public health researchers and practitioners working on diverse global health topics because of the simplicity of the estimation methods, low marginal cost of primary data acquisition, and availability of established measures of relative economic status in many public-use household surveys (e.g., those administered by the DHS Program, World Bank, International Labour Office, and UNICEF).

## METHOD

**FIGURE 1.**
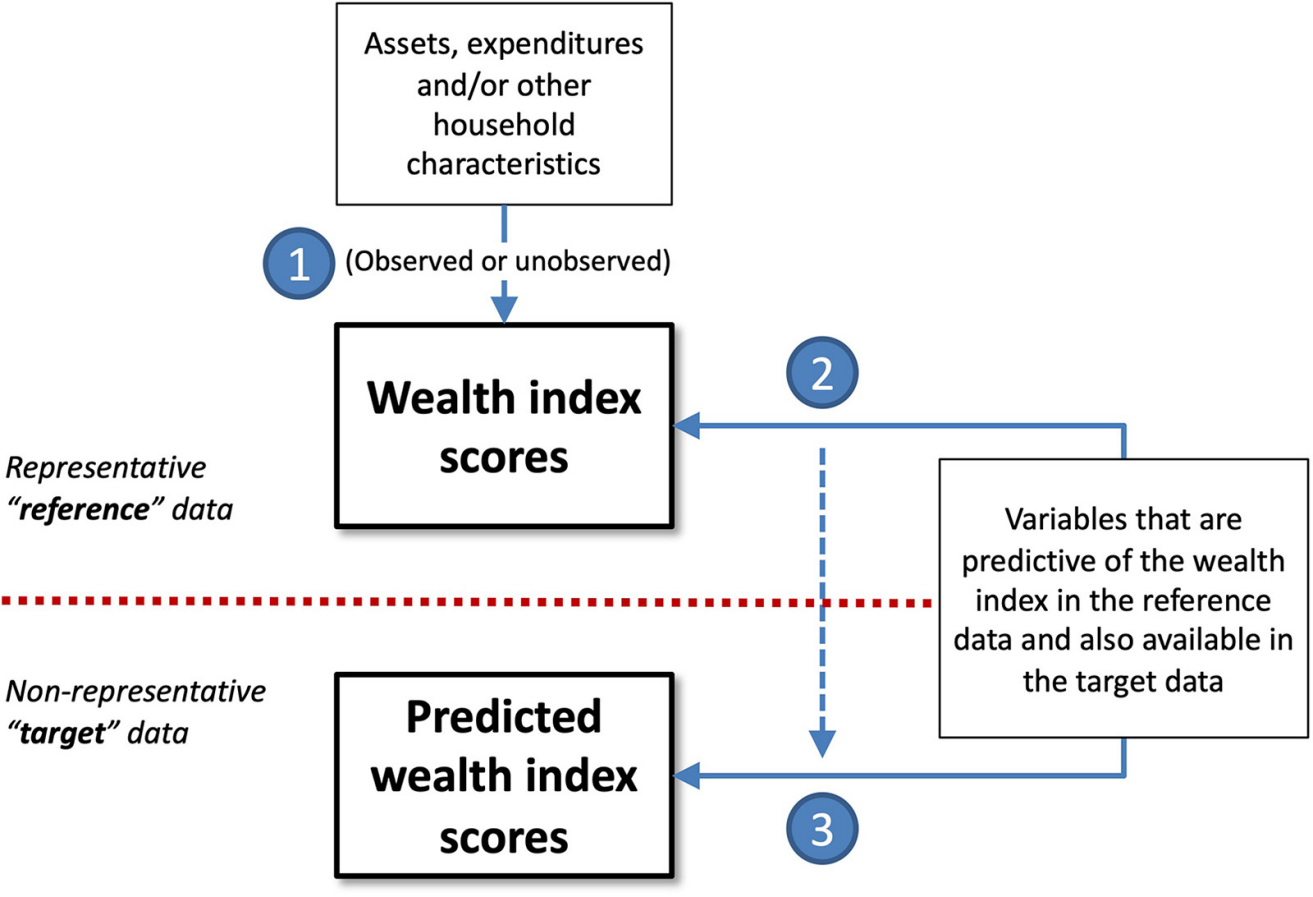
Overview of the Approach for Estimating the Relative Economic Status of Small and Nonrepresentative Samples

The approach for estimating relative economic position in small and nonrepresentative samples consists of 3 steps (Figure 1), which are broadly outlined below and discussed in further detail in Supplement 1. The implementation of the approach is described in 2 sample applications.

### Step 1: Define a Measure of Relative Economic Status in the Reference Data

First, a continuous measure of relative economic status must be identified or constructed in a representative reference dataset. Hereinafter, we refer to this measure as a “wealth index,” though other measures may be used, such as actual household wealth, household income or earnings, or consumption. The derivation of the wealth index may be observed or unobserved by the practitioner; it is not necessary that all components of the index or the detailed methods for its development are known. Instead, the approach relies on the index’s correlations with the unobserved construct it is intended to describe (e.g., household wealth or economic status) and with variables observed in both the reference and target data.

### Step 2: Model the Wealth Index in the Reference Data

This step involves the development of a model that provides a good fit of the wealth index in the reference data using only variables that are available in both the reference and target data. Using ordinary least squares regression methods, we estimate the association between the wealth index scores as the dependent variable and a variety of household assets and other characteristics as covariates. The parameter estimates from the regression model describe the incremental contribution of each household characteristic to household wealth.

### Step 3: Apply the Model Estimates to the Target Data

In the last step, data on household characteristics in the reference population and the estimated contribution of each characteristic to household wealth are combined to generate out-of-sample predictions of household wealth in the target sample. Specifically, a predicted wealth index score is generated for each household as a linear combination of household characteristics and the corresponding parameter estimates from the regression model developed in Step 2. This prediction approach is comparable to scoring methods applied in the context of proxy means testing (e.g., to assess program eligibility,^30^^–^^32^ develop poverty scorecards,^33^ track changes in poverty over time,^34^^,^^35^ or generate propensity scores for matching purposes^36^^–^^40^).

### Applicability and Relevance

Pertinent applications of the approach include comparing the characteristics of program beneficiaries or research participants to potentially eligible reference populations to, for example, characterize inclusion or exclusion errors in the targeting of social assistance programs, describe selection effects in the use of facility-based services, or assess selection biases in research studies. Results provide information on similarities, differences, and/or disparities in the distributions of relative economic status using a single metric, which may be further explored using other quantitative, qualitative, and/or anthropological methods.

The validity of the approach relies on the assumption that the associations between the characteristics evaluated in the model and the construct they are meant to describe are similar in the reference and target populations. The validity of this assumption, and the optimal choice of reference data, may vary across populations and over time and should be evaluated on a case-by-case basis. To evaluate the applicability in a specific context, researchers and evaluators should consider whether their target sample is captured by or in some way represented within the reference sample and whether the characteristics included in the model are equally relevant to the reference and target populations. Stratification of the reference data to more homogenous or relevant subpopulations—including, for example, regional or demographic subsamples—may increase the probability that the associations observed in the reference population are applicable to the respective target population.

The approach’s validity relies on the assumption that the associations between the characteristics evaluated in the model and the construct they are meant to describe are similar in the reference and target populations.

The approach can be equally applied to individual- and household-level data, though a household-level index is often the most relevant metric even when the target sample comprises individuals (e.g., adults or children). This is because many assets are shared across household members, and many household characteristics (e.g., characteristics of the dwelling and availability of common or shared assets) correlate with household composition. In fact, individuals are often asked about household characteristics, including in household surveys where a single reference person answers questions on behalf of the household. If a household-level index is estimated, then the characteristics and the predicted wealth index scores will apply to all members of the household. In sample surveys, survey-analytic weights should be appropriately incorporated into the estimation of the wealth index models.

One important exception to the applicability of the approach would be programs that target unhoused populations. In this case, one might question whether a household-based survey (e.g., DHS) is an appropriate reference sample and whether the DHS wealth index, which includes many housing characteristics, is applicable in this context. Similar challenges may be confronted if targeting an institutionalized population.

### Ethical Approval

The study protocols whose data are used in the sample applications received ethical approval from the institutional review boards at Duke University and the respective study sites: Meahto Phum Ko’mah (Battambang, Cambodia), SaveLives Ethiopia (Addis Ababa, Ethiopia), Sharan (Delhi, India), ACE Africa (Bungoma, Kenya), and Kilimanjaro Christian Medical Centre (Moshi, Tanzania), and regulatory agencies in all participating countries: National Ethics Committee for Health Research (Cambodia), Ministry of Science and Technology (Ethiopia), Indian Council of Medical Research (India), Kenya Medical Research Institute (Kenya), and the National Institute for Medical Research (Tanzania).

## SAMPLE APPLICATIONS USING DHS

To demonstrate the utility of the approach for characterizing the economic status of small and nonrepresentative populations, we describe 2 sample applications. In the first application, we describe the selection of a parsimonious model of the DHS wealth index and the effect of alternative model specifications on model fit. We subsequently compare the distribution of actual and predicted wealth index scores among urban households in Tanzania with the distributions of predicted wealth index scores among 2 urban samples of male and female participants in a research study related to HIV testing. In the second application, to demonstrate the method’s utility for both within- and cross-country comparisons, we apply the method to samples of households with orphaned and separated children in rural and urban settings in 5 LMICs. Both applications were implemented in Stata version 16.1 (StataCorp) and used the DHS wealth index as the reference metric for characterizing relative economic status. The reference populations comprise national rural and urban DHS samples; the sampling strategies and data collection procedures for the target populations have been previously described.^28^^,^^29^ In each sample application, we progress through the 3 steps outlined earlier; the detailed methods used in the 2 sample applications are summarized in the following sections.

### Step 1: Define a Measure of Relative Economic Status

We used the DHS wealth index as a measure of relative economic status in representative reference data. This article does not focus on the construction of the wealth index; relevant methods have been described elsewhere.^5^^,^^41^^,^^42^ In short, the DHS wealth index is derived using survey data from a nationally representative cluster sample of households. A variety of variables describing assets and other household characteristics are entered into a principal components analysis; variables’ loadings on the first component represent their contributions to the wealth index. The analysis typically includes 60 to 100 variables. A household’s wealth index score is calculated by multiplying loadings with the household’s values on each component variable and summing across variables.^42^ The wealth index is routinely derived for the DHS, UNICEF Multiple Indicator Cluster Surveys, Malaria Indicator Surveys, and AIDS Indicator Surveys and is assumed to be reflective of the distribution of wealth in the respective countries at the time of the survey.

### Step 2: Model the Wealth Index

Linear regression models were estimated with the DHS wealth index score as the dependent variable and various hypothesized correlates of household wealth as explanatory variables. Household-level DHS data from Cambodia (2010), Ethiopia (2011), India (2005–2006), Kenya (2008–2009), and Tanzania (2010 and 2015–2016) were used to estimate the contributions of each covariate to household wealth in relevant “reference” populations from each study site (Box).^43^

BOX Stata Commands Used to Calculate the Contribution of Each Covariate to the DHS Wealth Index

Covariates include variables that are available in both the reference data (i.e., the relevant DHS survey data) and the target data. In its simplest form, this step may be implemented in Stata using the following command:

. regress *wealthindex_reference predictor_variable_1 … predictor_variable_n*

In our sample applications, due to the complex survey design of the DHS reference data, the model was estimated using Stata’s *svy* command suite and relevant survey design variables:

. svyset *psu_variable*, strata(*strata_variable*) ‖ *household_id*, weight(*weight_variable*)

. svy: regress *wealthindex_reference predictor_variable_1 … predictor_variable_n*

To increase the probability that the associations observed in the reference population were applicable to the respective target population, models were estimated on geographic subsamples (i.e., rural vs. urban residence)^3^^,^^5^ of the available DHS data, using the subpop() option, for example:

. svy, subpop(*urban*): reg *wealthindex_reference predictor_variable_1 … predictor_variable_n*

In the selection of a parsimonious model, variables’ partial correlation coefficients and models’ R-squared statistics, root mean squared errors (RMSEs), and mean absolute errors in predicting households’ wealth index scores, wealth index quantiles, and rankings were used to compare the performance of alternative model specifications. This process is described in greater detail in Sample Application 1; the implementation is illustrated in Supplement 2.

### Step 3: Derive Wealth Index Scores

Parameter estimates from Step 2 were used to calculate a predicted wealth index score for every household in the respective “target” samples. Given that predictor variables are specified identically in the reference and target data, such out-of-sample predictions are particularly simple in Stata: . predict *pred_wealthindex_target*.

The distributions of actual and predicted wealth index scores in the reference and target samples are shown graphically. We used Student’s t-tests without the assumption of equal variance to assess the significance of differences in the means of predicted wealth index scores between the reference and target samples.

## SAMPLE APPLICATION 1: SELECTION OF A PARSIMONIOUS MODEL AND ESTIMATION OF WEALTH INDEX SCORES FOR 2 URBAN POPULATIONS AT HIGH RISK OF HIV INFECTION IN NORTHERN TANZANIA (2017–2018)

### Data

As part of the “Identifying and matching preferences for HIV/AIDS counseling and testing” study, 439 male Kilimanjaro mountain porters and 299 female barworkers—2 populations at elevated risk of HIV infection enrolled in an urban setting in Northern Tanzania—were asked about household assets and other characteristics. The sampling approach has been described elsewhere.^29^ Survey questions partially overlapped with questions in the 2015–2016 Tanzania DHS, allowing for a comparison of the economic status of the 2 target populations with the distribution of wealth index scores among reference urban households in Tanzania.

We compared economic status of 2 populations at elevated risk of HIV infection in urban Northern Tanzania with the distribution of wealth index scores among reference urban households in Tanzania.

### Model Estimation

We estimated survey regression models to describe the association between the DHS wealth index factor score and covariates among urban households. After the estimation of a “full” model with 19 covariates (available in both the reference and target data sets), 18 “reduced” models were estimated with the least informative variable, as indicated by the partial correlation coefficients, iteratively removed from the model. We assessed relative model performance using absolute and relative changes in models’ RMSEs, as well as deviations between the actual and predicted wealth index scores, percentiles, quintiles, and ranks, averaged across households in the reference sample. Parameter estimates from a parsimonious model with 14 covariates were used to calculate predicted wealth index scores for members of the 2 target samples. Annotated Stata code for model estimation—assessing model performance, predicting household wealth, and visualizing the wealth distributions in the reference and target samples—is shown in Supplement 2.

### RESULTS

**FIGURE 2.**
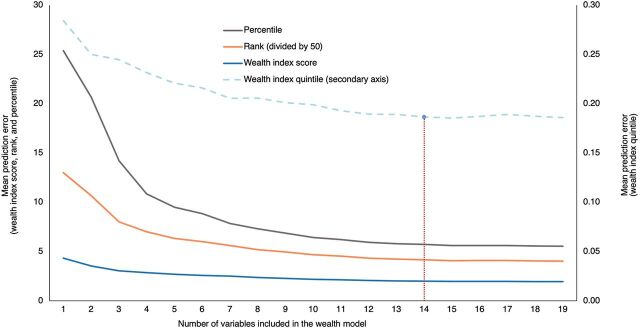
Mean Prediction Errors From Alternative Model Specifications: Limited Improvements in Models’ Predictive Ability From the Inclusion of Additional Covariates in the Wealth Model^a,b^ ^a^Supplement 2 provides information on the calculation. The Table provides information for the interpretation of prediction errors. ^b^The red dotted line indicates the selected (“reduced”) model with 14 covariates (Table).

**FIGURE 3.**
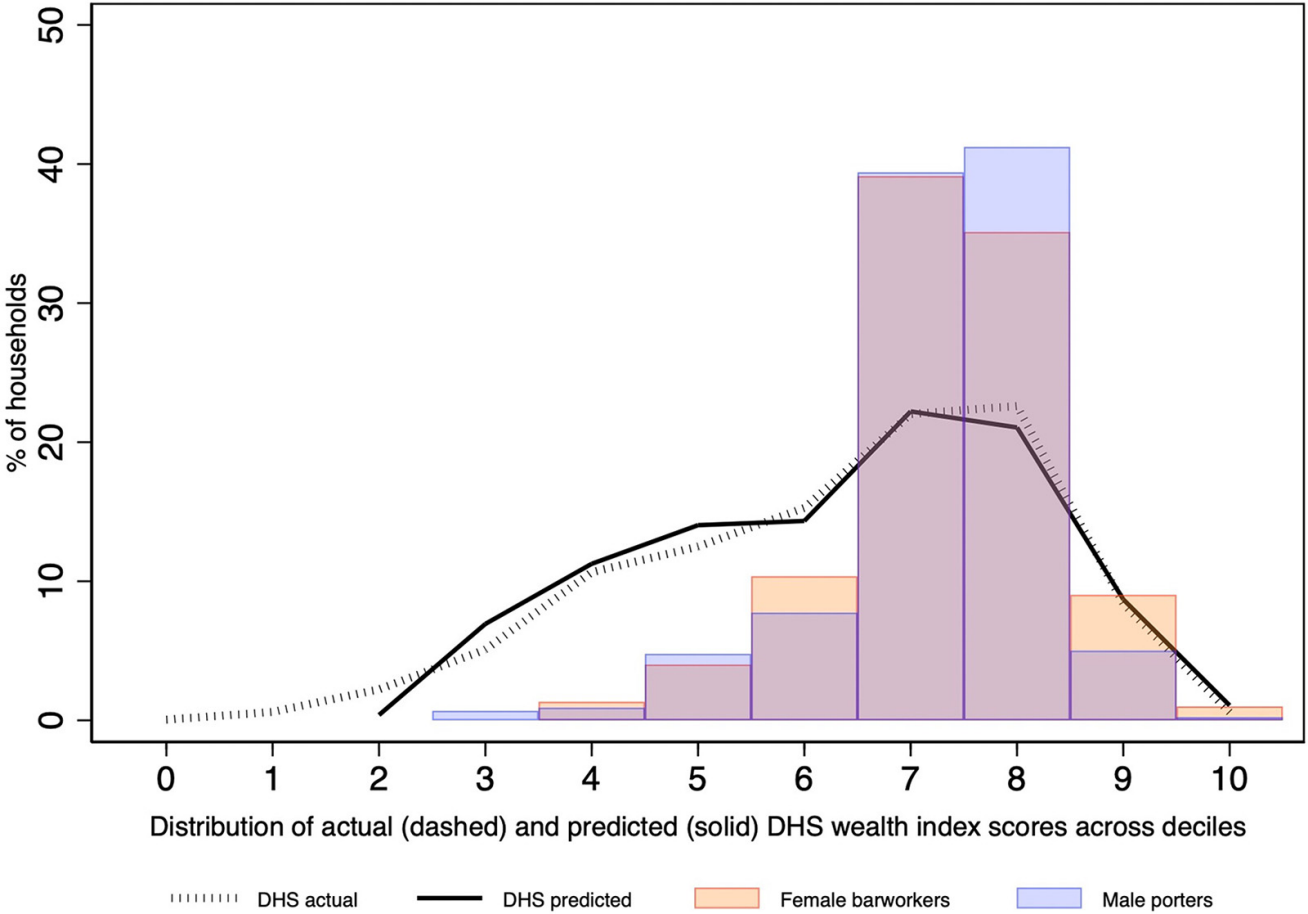
Distribution of Wealth Index Scores Among Urban DHS Households (N=3,634) and Predicted Wealth Index Scores Among Female Barworkers (N=299) and Male Porters (N=439) in Urban Northern Tanzania^a,b,c^ Abbreviation: DHS, Demographic and Health Survey. ^a^Data from the 2015–2016 Tanzania DHS and the “Identifying and matching preferences for HIV/AIDS counseling and testing” study. ^b^The x-axis represents equally sized “bins” of wealth index scores in the reference population, rescaled to range from 0 to 10. Solid bars: distribution of predicted wealth index scores among female barworkers and male porters. Lines: distribution of actual and predicted wealth index scores in the urban Tanzania DHS reference sample. ^c^Sample distributions across wealth quintiles for the reference and target samples are shown in Supplement 4.

All 19 variables were statistically significantly associated with household wealth (Supplement 3) and jointly explained 91.9% of the variation in household wealth in the DHS reference sample (Table). However, 5 variables with the lowest partial correlation coefficients only marginally improved model performance, as described by the RMSE, R-squared statistics, and mean absolute prediction errors. Exclusion of these variables from the model did not qualitatively alter the estimated coefficients on other correlates of household wealth (Table) or the prediction errors (Table and Figure 2). The model with 14 variables explained 91.6% of the variation in wealth index scores across urban Tanzanian households. Based on the predicted wealth index scores from the final model, the 2 target sample populations were, on average, significantly wealthier than the national urban reference population (Figure 3). This result is also illustrated in a comparison of the estimated distributions of the reference and target samples across DHS wealth quintiles (Supplement 4).

**TABLE.** Selection of a Parsimonious Model of the DHS Wealth Index Score (Tanzania 2015–2016 DHS, Urban Sample, N=3,634)

		Changes in Model Performance From the Incremental Addition of Covariates to the Wealth Index Model				Mean Prediction Error^a^				Estimated Wealth Index Component Weight	
Variables	Partial Correlation Coefficient	R- squared^b^	RMSE^c^	RMSE Change (absolute)	RMSE Change, %	Wealth Index Score^d^	Percentile	Quintile	Rank	Full Model	Reduced Model
Electricity	0.561	0.636	5.544			4.32	25.4	0.28	649.9	5.60	5.72
Floor material: natural	−0.621	0.757	3.956	−1.59	−28.6	3.53	20.7	0.25	533.7	−6.56	−6.73
Refrigerator	0.300	0.814	3.626	−0.33	−8.3	3.04	14.2	0.24	400.7	2.59	2.65
Flush toilet	0.296	0.835	3.387	−0.24	−6.6	2.86	10.9	0.23	349.9	1.84	1.90
Iron	0.261	0.850	3.233	−0.15	−4.5	2.69	9.5	0.22	316.0	1.78	1.78
Mobile phone	0.273	0.862	3.152	−0.08	−2.5	2.58	8.8	0.22	299.9	3.11	3.09
Livestock	−0.288	0.874	2.941	−0.21	−6.7	2.50	7.8	0.21	279.7	−1.92	−2.13
Computer	0.206	0.886	2.872	−0.07	−2.3	2.37	7.3	0.21	259.5	2.18	2.44
Television	0.252	0.894	2.767	−0.11	−3.7	2.26	6.9	0.20	247.7	2.18	2.20
Bank account	0.266	0.900	2.688	−0.08	−2.9	2.18	6.4	0.20	233.6	1.70	1.75
Agricultural land	−0.278	0.906	2.586	−0.10	−3.8	2.13	6.2	0.19	226.4	−1.77	−1.81
Car or truck	0.227	0.911	2.552	−0.03	−1.3	2.06	5.9	0.19	216.4	2.56	2.66
Radio	0.189	0.914	2.506	−0.05	−1.8	2.02	5.8	0.19	211.5	1.15	1.10
Persons per sleeping room	−0.143	0.916	2.473	−0.03	−1.3	1.99	5.7	0.19	207.8	−0.37	−0.34
Tapwater	0.111	0.916	2.461	−0.01	−0.5	1.96	5.6	0.19	203.6	1.17	
No. of rooms used for sleeping	−0.104	0.917	2.445	−0.02	−0.7	1.96	5.6	0.19	204.1	−0.29	
Treats drinking water	0.088	0.918	2.438	−0.01	−0.3	1.96	5.6	0.19	204.0	0.48	
Motorcycle or scooter	0.088	0.919	2.429	−0.01	−0.4	1.95	5.6	0.19	202.4	0.73	
Cooking fuel: electricity or gas	0.070	0.919	2.423	−0.01	−0.2	1.94	5.5	0.19	200.8	0.73	

Abbreviations: DHS, Demographic and Health Survey; RMSE, root mean squared error.

^a^Mean prediction errors were calculated as the sample averages of the absolute values of the actual minus predicted values of the respective measure of household wealth (wealth index, percentile, quintile, rank), meaning that they represent the average distance between the actual and predicted measures of household wealth across all urban DHS households. Supplement 2 has details on the calculation and implementation in Stata and Figure 2 provides a graphical representation of changes in prediction errors with the inclusion of additional covariates.

^b^Partial correlation coefficients were derived from a model that included all variables listed and a constant.

^c^R-squared and RMSE values, commonly used measures of model accuracy, are for the “accumulated” model (i.e., a model that includes the respective variable and all variables listed earlier in the table). RMSE change was calculated by comparing models with versus without the respective variable.

^d^Estimated wealth index component weights are the parameter estimates from the corresponding (full or reduced) multivariable linear regression model.

### Interpretation

All 19 variables correlated with the wealth index in the reference sample, and thus all variables could have been used to predict household wealth in the target samples. However, model performance improved only marginally after 14 variables, which highlights the value of the approach for prioritizing survey items needed to characterize household wealth in the target samples. While additional variables predictive of household wealth may improve prediction accuracy, the incremental improvements diminish with additional variables, reflecting a trade-off between prediction accuracy and the time and monetary costs associated with collecting additional survey data. Both statistical considerations and resource constraints, therefore, influence the optimal model specification.

The distribution of predicted wealth index scores in 2 nonrepresentative samples of men and women at above-average risk of HIV infection indicates that both samples were concentrated in the upper half of the wealth index score distribution among urban Tanzanian DHS households. Differences in the relative economic well-being of barworkers and porters may be a result of the selection of the samples from the Kilimanjaro Region (which is among the wealthiest regions in the country), employment-based sampling (e.g., only those with a job as a barworker or porter were eligible to participate), or self-selection into the 2 groups (e.g., those who are better educated may be more willing to engage with clients or participate in a research study).

## SAMPLE APPLICATION 2: ESTIMATION OF WEALTH INDEX SCORES FOR HOUSEHOLDS WITH ORPHANED AND SEPARATED CHILDREN IN 5 COUNTRIES (2006–2012)

### Data

As part of the Positive Outcomes for Orphans study,^28^ a stratified, cluster-randomized sample of 1,480 community-based orphaned and separated children was enrolled from 6 study sites in 5 LMICs, namely Cambodia, Ethiopia, India, Kenya, and Tanzania. Four of the 6 sites included both rural and urban samples. Sample sizes in the rural and urban settings in each study site with complete data on all relevant covariates ranged from 87 to 250.

### Model Estimation

For exemplification and comparability of estimates, we included in the models only variables that were common to all 5 DHS surveys as well as the Positive Outcomes for Orphans survey. Notably, this is not a requirement for cross-national comparisons; instead, researchers/evaluators should follow the model approach described in Sample Application 1 separately for each country and the respective rural and urban subsamples. In total, 11 variables were used as covariates in the survey regression models (Supplement 5). Separate models were estimated for rural and urban samples in each study site. The Stata code from Sample Application 1 (Supplement 2) is readily adapted to estimate distinct wealth index models and make separate predictions for rural and urban samples in each study site.

We estimated wealth index scores for rural and urban households with orphaned and separated children in 5 countries.

### Results

**FIGURE 4.**
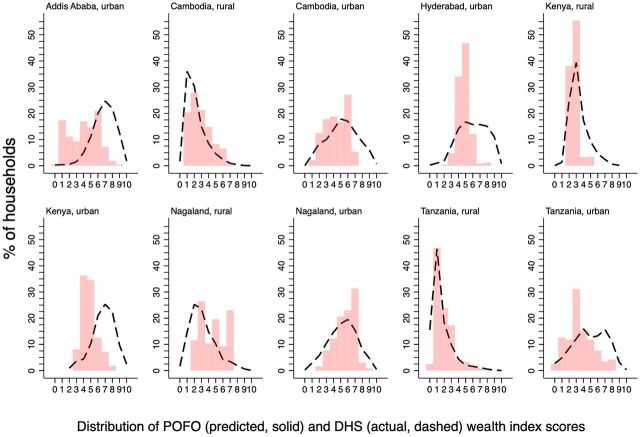
Distribution of Wealth Index Scores Among Households With Orphaned and Separated Children in 5 Countries^a,b^ Abbreviations: DHS, Demographic and Health Survey; POFO, Positive Outcomes for Orphans. ^a^Data from the POFO study and the corresponding DHS from each country. ^b^The x-axis represents equally sized “bins” of wealth index scores in the respective reference population, rescaled to range from 0 to 10. For visual clarity, the comparisons of actual vs. predicted wealth index scores for DHS households were omitted from this figure and are instead shown in Supplement 7. Solid red bars: distribution of predicted wealth index scores among households with orphaned and separated children. Dashed black lines: distribution of actual wealth index scores in the respective DHS reference samples.

All but 2 of the 109 evaluated associations between covariates and household wealth were statistically significant (Supplement 6). In 5 of 6 urban sites and 1 of 4 rural sites, wealthier households were under-represented among households caring for orphaned and separated children, compared with national reference populations (Figure 4). Conversely, in 1 urban site and 3 rural sites, households with orphaned and separated children were predicted to be wealthier than the relevant reference populations.

### Interpretation

Differences in the relative economic well-being of households caring for these children may be a result of the selection of the samples (e.g., from poorer or wealthier areas), specific characteristics of the orphan epidemic (e.g., affecting households with a higher vs. lower socioeconomic status), and/or cultural and economic determinants of the caregivers of these children after the loss of their parent (e.g., those with the means to do so).

## DISCUSSION

We describe a simple out-of-sample prediction approach to estimate measures of relative economic status for small and nonrepresentative samples. The method can be used to characterize the relative economic status of virtually any sample or population of interest, provided that comparable data on correlates of household wealth (or, alternatively, income or consumption) are available for both the target sample and an appropriate reference population. The approach minimizes uncertainty over how to weigh diverse household characteristics and concerns about the precision and external comparability of estimates derived from small and nonrepresentative samples.

The simplicity of the approach and low marginal cost of primary data acquisition suggest that the method may be broadly applicable and useful to researchers, implementers, and evaluators seeking to compare the characteristics of research participants or program beneficiaries to the general population. Chasekwa et al., for example, used a similar approach to compare the wealth among participants in the Sanitation, Hygiene, Infant Nutrition Efficacy (SHINE) trial to the general population in rural Zimbabwe (albeit in the context of validating a within-sample wealth index).^6^ While the sample applications presented in our article use DHS data, any nationally or regionally representative data source that includes information about household characteristics and economic position may be used as reference data. The public availability of data on expenditures in nationally representative consumption surveys commissioned by the World Bank^44^; assets, earnings, and expenditures in household surveys commissioned by ILO^45^; and wealth index scores in surveys supported by the DHS program^43^ make these surveys particularly useful sources of reference data, especially in LMICs. The increasing harmonization of survey tools across countries and over time suggests that the approach proposed here may also lend itself to demographic, geographic, and temporal comparisons of household wealth distributions across different reference data.

Our approach may be broadly applicable and useful to researchers, implementers, and evaluators seeking to compare the characteristics of research participants or program beneficiaries to the general population.

### Limitations

The approach is subject to several important limitations. Most importantly, all limitations of the measure of relative economic status in the reference data (e.g., wealth index) also apply to the estimated index scores in the target data. Specifically, the approach assumes that the relationship between the index and the construct it intends to measure, as well as their associations with each of the index’s components, are the same in both populations. The validity of this assumption and the optimal choice of reference data may vary across populations and over time and should be evaluated on a case-by-case basis. For example, the meaning of a mobile phone as an indicator of economic well-being has changed dramatically over time and may vary across populations. A household-level approach may not be appropriate if the objective is to characterize resource allocation across individuals within households. Counterintuitive associations may be observed due to the omission of relevant variables (e.g., missing measures on household composition when predicting the relative wealth of individuals). It is also possible that plausible correlates of wealth may not have been included in the design of the wealth index in the reference data, potentially leading to counterintuitive associations in models of the wealth index. For example, a negative association between landownership and household wealth was observed in the DHS. Improved methods for deriving wealth indices in the reference populations should reduce such “errors”; several adjustments to the DHS wealth index were made or proposed in recent years^42^ to improve the index’s underlying properties.

Second, the model of the wealth index is limited by the amount of overlap in potential covariates between the reference and target data. Thus, whenever possible, the method should be considered in a priori decisions regarding primary data collection for the target population so that all relevant wealth index component variables can be included in survey instruments. While any variables common to both reference and target data can be added to the model, standard goodness-of-fit tests should be used to ensure that only variables that are correlated with the wealth index are included. In the sample applications described above, high model R-squares and small incremental changes in RMSE and model performance metrics suggested that additional variables are not likely to substantively improve the estimated models.

Third, careful attention should be paid to the selection of an appropriate reference population, which may be composed of regional or demographic subsamples of the available reference data. Model stratification to more homogenous populations (e.g., households with or without children) or geographic restrictions (e.g., households in rural or urban areas) may increase the probability that the associations observed in the reference population are applicable to the respective target population but may limit the external validity of the models. In sample surveys, survey-analytic weights should be appropriately incorporated into the estimation of the wealth index models.

Finally, the predicted wealth index combines various sources of error, including measurement error (e.g., due to misreporting on covariates), estimation error (due to incorrect model specification), and prediction error (the index can only take on a finite number of values determined by the number of possible combinations of its component variables). If the index is to be used as an explanatory variable in other models, bootstrapping methods should be used to ensure that those models yield appropriate standard errors.

Despite these limitations, the approach described here offers a novel and widely applicable tool for public health researchers and practitioners seeking to compare small and nonrepresentative samples of research participants or program beneficiaries to the general population. Such information may be used, for example, to better characterize and eventually mitigate inclusion and exclusion errors for social assistance programs, access barriers to facility-based services, or selection biases in research studies.

## CONCLUSION

We describe a method for estimating wealth index scores and characterizing the economic status of small and nonrepresentative samples. The simple analytic approach, readily available reference data, and low marginal cost of primary data acquisition suggest that this method may be useful to public health professionals who implement, manage, and evaluate programs in low- and middle-income countries.

## Supplementary Material

GHSP-D-22-00394-supplement.pdf

## Notes

**Peer Reviewed**

**First published online:** April 4, 2023.
