# Supplementary material for: How Poor Is Your Sample? A Simple Approach for Estimating the Relative Economic Status of Small and Nonrepresentative Samples
Source: Glob Health Sci Pract. 2023 Apr 28;11(2):e2200394. doi: 10.9745/GHSP-D-22-00394 (PMC10141430; doi:10.9745/GHSP-D-22-00394)
Supplement: GHSP-D-22-00394-supplement.pdf [file GHSP-D-22-00394-supplement.pdf]

Supplement to: Ostermann J, Hair N, Grzimek V, et al. How poor is your sample? A simple approach for estimating the relative economic status of small and nonrepresentative samples. *Glob Health Sci Pract*. 2023;11(2):e2200394. https://doi.org/10.9745/GHSP-D-22-00394

Supplement 1. Formal description of the estimation and prediction approach

Let $\widehat{W}_i$ describe the estimated wealth index score of household i, defined as a linear combination (or some transformation of a linear combination) of a vector of household characteristics $\boldsymbol{x_i}$ and their average contributions (or weights) $\boldsymbol{w}$ to household wealth.

$$\widehat{W}_i = \sum_{k=1}^{K} w_k\, x_{ki}$$

The vector $\boldsymbol{w}$ is commonly derived from some reference population either by regressing household characteristics on economic indicators such as expenditures, or using principal components analysis, though other approaches to weighting exist. Under the assumption that the associations between components of the index and the construct it intends to measure (e.g., household wealth) are the same in both populations, weights derived from one population represent unbiased estimates of $\boldsymbol{\hat{w}}$ for the other, and thus can be applied to calculate a wealth index score $\widehat{W}_j$ for each household j in the target population.

$$\widehat{W}_j = \boldsymbol{\hat{w} x_j}$$

If the weights of all components of the wealth index in the reference data were known, and if all components were equally observed in the reference and target populations, the wealth index could simply be calculated for each household j in the target population using the vector $\boldsymbol{w}$. However, if the weights are not known, or not all variables comprising the index are observed in the target data, the weights need to be estimated. The next step therefore involves the development of a model that provides a good fit of the wealth index in the reference data but includes only variables available in both the reference and target data. The vector of parameter

estimates $\boldsymbol{\beta}$ represents an estimate of $\hat{\boldsymbol{w}}$, i.e., the contribution of each covariate to household wealth $\widehat{W}$.

In the last step, data on household characteristics and the estimated contribution of each characteristic to household wealth are combined to generate an out-of-sample prediction of household wealth $\widehat{W}_j$ for each household j in the target sample. Specifically, a wealth index score $\widehat{W}_j$ can be generated for each household j as a linear combination of characteristics $\boldsymbol{x_j}$ and the corresponding parameter vector $\boldsymbol{\beta}$ derived in step 2:

$$\widehat{W}_j = \sum_{k=1}^{K} \beta_k \, x_{kj}.$$

Supplement to: Ostermann J, Hair N, Grzimek V, et al. How poor is your sample? A simple approach for estimating the relative economic status of small and nonrepresentative samples. *Glob Health Sci Pract*. 2023;11(2):e2200394. https://doi.org/10.9745/GHSP-D-22-00394

Supplement 2. Stata code for all key steps outlined in Sample Application 1

```
*Note: With the appropriate specification of the data file and path names, the code shown below should run in Stata as-is
```

/** REFERENCE DATA - TANZANIA - DHS 7 **/

```
*identify candidate variables in the DHS data documentation; specifying these variables here avoids limitations with the maximum number of variables in certain Stata versions

global KEEPVARS hhid hv000 hv001 hv002 hv005 hv004 hv021 hv022 hv024 hv025 hv201 hv205 hv206 hv207 hv208 hv209 hv210 hv211 hv212 hv213 hv216 hv237 hv243e hv244 hv246 hv247 sh121h hv243a hv041 hv009 hv270 hv270a hv271a hv271 hv270a hv271a hv226 hv214 hv215 sh115 hv243c
```

```
*Specify path to the data
global DHSPATH "/Users/../"
global WORKPATH "/Users/../tmp"
cd $WORKPATH

use $KEEPVARS using "$DHSPATH/TZHR7HFL.DTA", clear
```

/** recode potential correlates of wealth as covariates for the model **/

```
   /* ASSETS */
      capture program drop doit
      program define doit
         local result=lower("`2'")
         ren `1' `result'
         global assetlist $assetlist `result'
      end

      global assetlist
       doit hv206  "Electricity"
       doit hv207  "Radio"
       doit hv208  "Television"
       doit hv209  "Refrigerator"
       doit hv210  "Bicycle"
       doit hv211  "Motorbike"
       doit hv212  "Car"
       doit sh121h "Iron"
       doit hv243e "Computer"
       doit hv243a "Mobilephone"
       doit hv247  "Bankaccount"
       doit hv244  "AgricultLand"
       doit hv246  "Livestock"
       doit hv243c "Animalcart"
```

Supplement to: Ostermann J, Hair N, Grzimek V, et al. How poor is your sample? A simple approach for estimating the relative economic status of small and nonrepresentative samples. *Glob Health Sci Pract*. 2023;11(2):e2200394. https://doi.org/10.9745/GHSP-D-22-00394

```
    /* WATERSOURCE */
        gen tapwater=hv201==11


    /* TOILET */
        gen flushtoilet=inlist(hv205,11,12,13,14,15)


    /* PERSONS PER BEDROOM */
        gen rooms=hv216
        gen perbedroom=hv009/rooms
        replace perbedroom=1 if perbedroom<1


    /* FLOOR */
        gen badfloor=0 if inlist(hv213,30,31,32,33,34,35,96)
        replace badfloor=1 if inlist(hv213,10,11,12,20,21,22)


    /* Treats water */
        gen treatswater=hv237==1


    /* Cooking fuel */
        gen cookingfuel = inlist(hv226,1,12)


    /* WEALTH INDEX */
        *continuous factor score
        gen wealthindexf=hv271/10000
        *quintiles
        gen wealthindex=hv270


    /* SAMPLING WEIGHT */
        replace hv005=hv005/1000000


    /* URBAN */
        gen urban=hv025==1


keep hhid hv000 hv001 hv002 hv005 hv004 hv021 hv022 hv024 urban tapwater flushtoilet
perbedroom wealthindex* badfloor $assetlist treatswater rooms cookingfuel

compress


save "$WORKPATH/reference_sample", replace
```

Supplement to: Ostermann J, Hair N, Grzimek V, et al. How poor is your sample? A simple approach for estimating the relative economic status of small and nonrepresentative samples. *Glob Health Sci Pract*. 2023;11(2):e2200394. https://doi.org/10.9745/GHSP-D-22-00394

/** MODEL DEVELOPMENT **/

```
use "$WORKPATH/reference_sample", clear

*List of candidate variables
global INDVARS electricity badfloor refrigerator flushtoilet iron mobilephone
livestock computer television bankaccount agricultland car radio perbedroom tapwater
rooms treatswater motorbike cookingfuel


   *get partial correlation coefficients
   pcorr wealthindexf $INDVARS  if urban==1 [aw=hv005]


   *Estimate the full model
   svyset hv021, strata(hv022) || hhid, weight(hv005)
   svy, subpop(urban): reg wealthindexf $INDVARS


   *get RMSE for different models
      reg wealthindexf electricity [aw=hv005]
      di "RMSE = " e(rmse)

      reg wealthindexf electricity badfloor [aw=hv005]
      di "RMSE = " e(rmse)

      *... continue with other models

      reg wealthindexf $INDVARS [aw=hv005]
      di "RMSE = " e(rmse)


   *get R^2 for different models
      svy, subpop(urban): reg wealthindexf electricity
      di "R^2 = " e(r2)

      svy, subpop(urban): reg wealthindexf electricity badfloor
      di "R^2 = " e(r2)

      *... continue with other models

      svy, subpop(urban): reg wealthindexf $INDVARS
      di "R^2 = " e(r2)


   pause: identify your preferred model based on changes in RMSE and R2
```

Supplement to: Ostermann J, Hair N, Grzimek V, et al. How poor is your sample? A simple approach for estimating the relative economic status of small and nonrepresentative samples. *Glob Health Sci Pract*. 2023;11(2):e2200394. https://doi.org/10.9745/GHSP-D-22-00394

/** ACTUAL AND PREDICTED VALUES FOR PREFERRED MODEL **/

```
global INDVARS electricity badfloor refrigerator flushtoilet iron mobilephone
livestock computer television bankaccount agricultland car radio perbedroom

   use "$WORKPATH/reference_sample", clear
   svy, subpop(urban): reg wealthindexf $INDVARS
   estimates store wealth_model
```

*Predictions (limit to estimation sample)

```
      keep if e(sample)

      *wealth index factor score
      gen wealthindexf_act=wealthindexf
      predict wealthindexf_pred, xb

      *percentiles
      xtile pctile_act  = wealthindexf_act, nq(100)
      xtile pctile_pred = wealthindexf_pred, nq(100)

      *quintiles: get cutoff values from the full dataset:
      * . ta wealthindex, c(min wealthindexf max wealthindexf)
      gen quintile_act      = wealthindex
      gen quintile_pred     = 1 if wealthindexf_pred<-8.7643
      replace quintile_pred = 2 if inrange(wealthindexf_pred,-8.7643,-6.6276)
      replace quintile_pred = 3 if inrange(wealthindexf_pred,-6.6276,-2.6220)
      replace quintile_pred = 4 if inrange(wealthindexf_pred,-2.6220, 8.7222)
      replace quintile_pred = 5 if inrange(wealthindexf_pred, 8.7222, 1000000)

      *rankings
      egen rank_act=rank(wealthindexf_act), unique
      egen rank_pred=rank(wealthindexf_pred), unique
```

*Prediction Errors

```
      *factor scores
       gen diff_wi = wealthindexf_pred-wealthindexf_act
      *percentiles
       gen diff_pctile = pctile_pred-pctile_act
      *quintiles
       gen diff_quintile = quintile_pred-quintile_act
      *rankings
       gen diff_rank = rank_pred-rank_act

      *absolute values of all differences (see Table 1)
       for var diff*: gen absX=abs(X)
       sum absdiff*
```

*Graph error distributions

Supplement to: Ostermann J, Hair N, Grzimek V, et al. How poor is your sample? A simple approach for estimating the relative economic status of small and nonrepresentative samples. *Glob Health Sci Pract*. 2023;11(2):e2200394. https://doi.org/10.9745/GHSP-D-22-00394

```
	for var diff*: dotplot X, name(X)
	graph combine diff_wi diff_pctile diff_quintile diff_rank
```

/** OUT-OF-SAMPLE PREDICTIONS **/

```
   pause: make sure you choose the right estimation model for predictions
   estimates restore wealth_model
   estimates replay wealth_model

   *apply component weights to the corresponding variables in the target data
   *Make sure that variable names and definitions are identical to those specified
    *in the wealth model that was estimated using the reference data

   use  "$WORKPATH/target_sample", clear
   capture drop target_wealthindexf_pred
   predict target_wealthindexf_pred
   save, replace
```

/** VISUALIZE DISTRIBUTIONS **/

```
	*number of buckets/bins for visualization of distributions
	global BINS 10
```

*Reference sample: Distribution of actual and predicted scores

```
	use "$WORKPATH/reference_sample", clear
	predict reference_wealthindexf_pred if urban==1
	keep if reference_wealthindexf_pred<.

	qui sum wealthindexf
	local wi_min=r(min)
	local xtile=(r(max)-r(min)) / $BINS

	gen xtile = round( (wealthindexf-r(min)) / `xtile',1 )
	gen xtile_pred = round( (reference_wealthindexf_pred-r(min)) / `xtile',1 )

	count
	gen N=r(N)
	gen one=1

	*Distribution of actual (rescaled) WI across wealth bins
	preserve
		collapse (sum) share_actual=one (mean) N, by(xtile)
		replace share_actual=share_actual/N*100
		tempfile actual
		save `actual', replace
	restore

	*Distribution of predicted (rescaled) WI across wealth bins
	preserve
		collapse (sum) share_pred=one (mean) N , by(xtile_pred)
```

```
            replace share_pred=share_pred/N*100
            tempfile pred
            save `pred', replace
        restore
```

Supplement to: Ostermann J, Hair N, Grzimek V, et al. How poor is your sample? A simple approach for estimating the relative economic status of small and nonrepresentative samples. *Glob Health Sci Pract*. 2023;11(2):e2200394. https://doi.org/10.9745/GHSP-D-22-00394

*Target sample: Distribution of predicted wealth index scores

```
use  "$WORKPATH/target_sample", clear
keep if target_wealthindexf_pred<.
gen xtile_target = round( (target_wealthindexf_pred-`wi_min') / `xtile',1 )

count
gen N=r(N)
gen one=1
preserve
	collapse (sum) share_target=one (mean) N , by(xtile_target)
	replace share_target=share_target/N*100
	tempfile target
	save `target', replace
restore
```

*Graph distributions across wealth bins

```
*install mmerge if needed
net install mmerge, from(http://fmwww.bc.edu/RePEc/bocode/m)

use `actual', clear
mmerge xtile using `pred', t(1:1) umatch(xtile_pred)
mmerge xtile using `target', t(1:1) umatch(xtile_target)

gen country="Tanzania, urban households:  2015/16 DHS vs. Target sample"

twoway line share_pred xtile,  lcolor(black) lw(medthick) || line share_actual
xtile, lcolor(black) lpattern(dot) lwidth(thick)  || bar share_target xtile,
color(orange%35) lcolor(red%50) lwidth(vthin) xlab(0(1)10) ylab(0(10)50)
xtitle("Distribution of actual (dashed) and predicted (solid) DHS wealth index
scores across deciles", size(small) margin(small)) xmtick(0(1)10) xla(0(1)10)
ytitle("% of households", size(small)) scheme(s1color) legend(order(2 "DHS
actual" 1 "DHS predicted" 3 "Target sample") rows(1) region(lc(white))
symxsize(*.5) symysize(*.6) size(*.6)) name(dhs_target, replace)
saving(dhs_target, replace)

exit
```

Supplement to: Ostermann J, Hair N, Grzimek V, et al. How poor is your sample? A simple approach for estimating the relative economic status of small and nonrepresentative samples. *Glob Health Sci Pract*. 2023;11(2):e2200394. https://doi.org/10.9745/GHSP-D-22-00394

2015-

Supplement 3. Correlates of the DHS wealth index in the 2016 Tanzania *Demographic and Health Survey (T-DHS)* and characteristics of participants in the 2017-18 *Identifying and Matching HIV/AIDS Counseling and Testing (IMPACT)* study

	Characteristics of the Urban T-DHS sample		Wealth index model with all covariates			Characteristics of Female Barworkers		Characteristics of Male Porters	
Variables	Mean	Std. Err	Coeff.		Std. Err.	Mean	Std. Err	Mean	Std. Err
Wealth index	10.12	(0.51)							
Electricity	0.56	(0.02)	5.60	***	(0.17)	0.90	(0.31)	0.34	(0.28)
Floor material: natural	0.17	(0.02)	-6.56	***	(0.30)	0.02	(0.14)	0.15	(0.80)
Refrigerator	0.23	(0.02)	2.59	***	(0.11)	0.32	(0.47)	0.38	(0.00)
Flush toilet	0.43	(0.02)	1.84	***	(0.15)	0.39	(0.49)	0.47	(0.06)
Iron	0.45	(0.02)	1.78	***	(0.13)	0.72	(0.45)	0.49	(0.00)
Mobile phone	0.92	(0.01)	3.11	***	(0.33)	0.99	(0.12)	0.10	(0.58)
Livestock	0.30	(0.02)	-1.92	***	(0.19)	0.16	(0.37)	0.38	(0.63)
Computer	0.11	(0.01)	2.18	***	(0.15)	0.06	(0.23)	0.29	(0.07)
Television	0.47	(0.02)	2.18	***	(0.15)	0.66	(0.47)	0.45	(0.10)
Bank account	0.64	(0.01)	1.70	***	(0.16)	0.42	(0.49)	0.45	(0.00)
Agricultural land	0.30	(0.02)	-1.77	***	(0.18)	0.09	(0.29)	0.30	(0.65)
Car or truck	0.09	(0.01)	2.56	***	(0.20)	0.06	(0.24)	0.16	(0.02)
Radio	0.63	(0.01)	1.15	***	(0.13)	0.78	(0.41)	0.28	(0.00)
Persons per sleeping room	2.33	(0.03)	-0.37	***	(0.06)	1.99	(0.92)	1.06	(0.01)
Tapwater	0.07	(0.01)	1.17	***	(0.16)				
# of rooms used for sleeping	2.05	(0.03)	-0.29	*	(0.12)				
Treats drinking water	0.53	(0.02)	0.48	***	(0.14)				
Motorcycle or scooter	0.12	(0.01)	0.73	***	(0.13)				
Cooking fuel: electricity or gas	0.09	(0.01)	0.73	***	(0.19)				
Constant			1.81	***	(0.35)				
Number of observations	3,632					299		439	

Notes: Estimated means or regression coefficients, with standard errors in parentheses. Regression coefficients estimated using a survey regression model with the DHS wealth index factor score as the dependent variable and continuous (persons per sleeping room, # of rooms used for sleeping) and binary indicator variables (all other household characteristics) as explanatory variables. *, **, and *** indicate statistical significance at the 0.05, 0.01, and 0.001 levels, respectively. Std. Err. – Standard Error; Coeff. – Coefficient

Supplement to: Ostermann J, Hair N, Grzimek V, et al. How poor is your sample? A simple approach for estimating the relative economic status of small and nonrepresentative samples. *Glob Health Sci Pract*. 2023;11(2):e2200394. https://doi.org/10.9745/GHSP-D-22-00394

Supplement 4. Comparison of the sample distributions across wealth quintiles for the reference and target samples

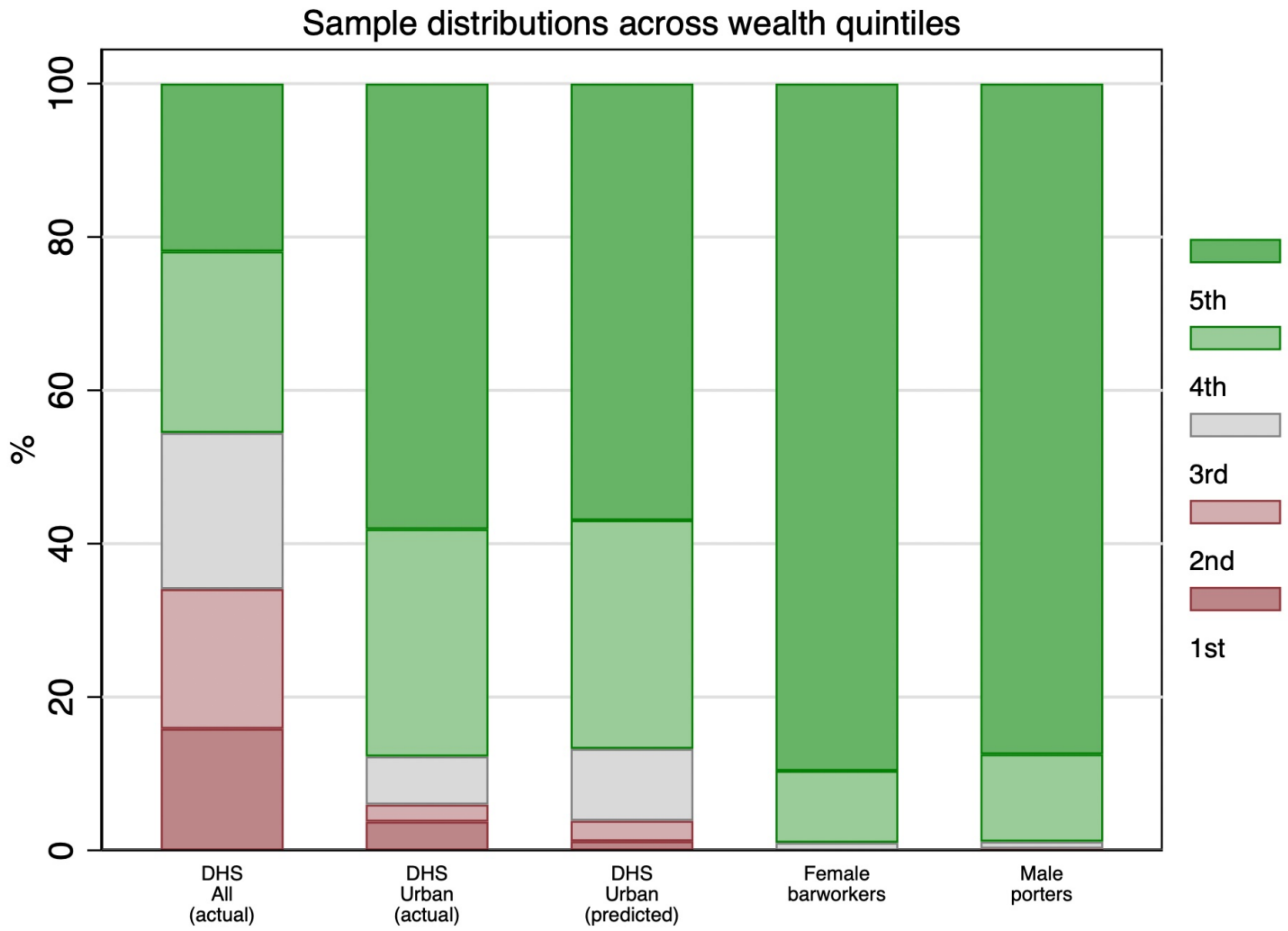

Notes: For comparability the percentages shown do not account for Demographic and Health Survey sampling weights.

Supplement to: Ostermann J, Hair N, Grzimek V, et al. How poor is your sample? A simple approach for estimating the relative economic status of small and nonrepresentative samples. *Glob Health Sci Pract*. 2023;11(2):e2200394. https://doi.org/10.9745/GHSP-D-22-00394

Supplement 5. Characteristics of DHS households included in the estimation of the wealth index reference models

Country	Cambodia	Addis Ababa	Hyderabad	Kenya	Nagaland	Tanzania
Year of survey	2010	2011	2005-06	2008	2005-06	2010
# of observations	15,667	5,112	2,741	9,057	3,866	9,623
Household characteristics						
Electricity	38.2%	98.6%	97.7%	25.0%	86.8%	15.0%
Radio	43.8%	82.3%	27.2%	71.2%	30.9%	61.2%
Television	61.6%	72.0%	79.2%	28.7%	45.7%	12.8%
Refrigerator	6.5%	32.4%	41.5%	8.4%	14.4%	6.8%
Motorbike	54.8%	0.2%	40.2%	2.1%	6.1%	3.7%
Car	8.1%	7.7%	9.7%	7.0%	7.1%	1.9%
Phone	64.8%	89.8%	56.2%	60.2%	28.6%	47.4%
Flush toilet	41.9%	10.9%	95.6%	16.6%	66.9%	10.1%
# of people per bedroom	3.93	2.68	3.40	2.75	2.61	2.48
Rudimentary / natural floor	78.4%	13.5%	42.0%	55.0%	58.2%	66.1%
Rudimentary / natural roof	14.2%	1.2%	1.5%	90.5%	18.1%	39.6%
Urban residence	28.0%	100.0%	100.0%	32.1%	48.8%	23.0%
DHS wealth index	3.08	4.98	4.39	3.25	3.43	3.01

Supplement to: Ostermann J, Hair N, Grzimek V, et al. How poor is your sample? A simple approach for estimating the relative economic status of small and nonrepresentative samples. *Glob Health Sci Pract*. 2023;11(2):e2200394. https://doi.org/10.9745/GHSP-D-22-00394

Supplement 6. Correlates of the DHS wealth index in five *Demographic and Health Surveys (DHS)* and the *Positive Outcomes for Orphans (POFO)* study

	Cambodia		Ethiopia	Hyderabad	Kenya		Nagaland		Tanzania	
**Household characteristics**	Rural	Urban	Urban	Urban	Rural	Urban	Rural	Urban	Rural	Urban
Electricity	4.29**	3.03**	9.85**	1.93**	4.98**	3.94**	0.82**	0.89**	7.35**	6.51**
	(0.07)	(0.21)	(0.31)	(0.32)	(0.18)	(0.20)	(0.15)	(0.24)	(0.15)	(0.18)
Radio	0.78**	1.73**	1.61**	1.25**	1.63**	2.09**	0.98**	1.18**	1.60**	2.60**
	(0.05)	(0.12)	(0.22)	(0.14)	(0.09)	(0.18)	(0.14)	(0.17)	(0.05)	(0.13)
Television	1.89**	2.42**	4.20**	3.11**	1.45**	1.26**	2.52**	2.84**	4.26**	3.47**
	(0.06)	(0.20)	(0.21)	(0.16)	(0.13)	(0.18)	(0.17)	(0.18)	(0.16)	(0.19)
Refrigerator	6.71**	6.47**	4.51**	4.04**	2.19**	2.56**	2.55**	3.51**	5.12**	3.81**
	(0.27)	(0.17)	(0.21)	(0.16)	(0.40)	(0.22)	(0.28)	(0.21)	(0.22)	(0.17)
Motorbike	1.29**	2.20**		2.56**	0.90**	1.32**	1.81**	2.03**	2.91**	2.50**
	(0.05)	(0.15)		(0.15)	(0.32)	(0.37)	(0.32)	(0.28)	(0.15)	(0.23)
Car	4.40**	4.76**	3.06**	3.61**	0.69**	2.37**	3.22**	3.32**	4.49**	3.87**
	(0.13)	(0.17)	(0.33)	(0.22)	(0.26)	(0.23)	(0.41)	(0.26)	(0.27)	(0.26)
Phone	1.99**	2.30**	2.85**	4.17**	2.24**	3.84**	3.73**	3.41**	2.23**	4.63**
	(0.06)	(0.20)	(0.29)	(0.16)	(0.09)	(0.19)	(0.23)	(0.19)	(0.05)	(0.14)
Flush toilet	3.65**	4.91**	2.09**	0.52	4.69**	4.09**	1.48**	1.38**	3.25**	2.88**
	(0.06)	(0.18)	(0.28)	(0.29)	(0.32)	(0.17)	(0.13)	(0.19)	(0.14)	(0.13)
# of people per bedroom	-0.55**	-0.68**	-0.23**	-0.50**	-0.46**	0.16**	-0.65**	-0.57**	-0.16**	0.28**
	(0.01)	(0.03)	(0.05)	(0.04)	(0.02)	(0.04)	(0.03)	(0.05)	(0.02)	(0.04)
Rudimentary / natural floor	-6.79**	-5.73**	-4.36**	-2.64**	-6.39**	-5.15**	-6.34**	-4.40**	-5.45**	-5.14**
	(0.06)	(0.12)	(0.24)	(0.13)	(0.10)	(0.21)	(0.14)	(0.17)	(0.06)	(0.15)
Rudimentary / natural roof	-2.13**	-2.94**	-6.77**	-7.72**	-1.97**	-0.15	-2.78**	-3.71**	-3.01**	-5.23**
	(0.07)	(0.32)	(0.77)	(0.50)	(0.12)	(0.15)	(0.16)	(0.25)	(0.05)	(0.24)
Observations	11,221	4,365	1,493	2,735	6,111	2,888	1,977	1,888	7,340	2,190
R-squared	0.89	0.93	0.97	0.92	0.86	0.93	0.88	0.84	0.93	0.97

Note: Regression coefficients (with standard errors in parentheses) were estimated using a survey regression model with the DHS wealth index factor score as the dependent variable and continuous (persons per sleeping room, # of rooms used for sleeping) and binary indicator variables (all other household characteristics) as explanatory variables. *, **, and *** indicate statistical significance at the 0.05, 0.01, and 0.001 levels, respectively. The coefficient for Motorbike in Ethiopia was not estimable.

Supplement to: Ostermann J, Hair N, Grzimek V, et al. How poor is your sample? A simple approach for estimating the relative economic status of small and nonrepresentative samples. *Glob Health Sci Pract*. 2023;11(2):e2200394. https://doi.org/10.9745/GHSP-D-22-00394

Supplement 7. Distributions of actual and predicted DHS wealth indices across POFO study sites

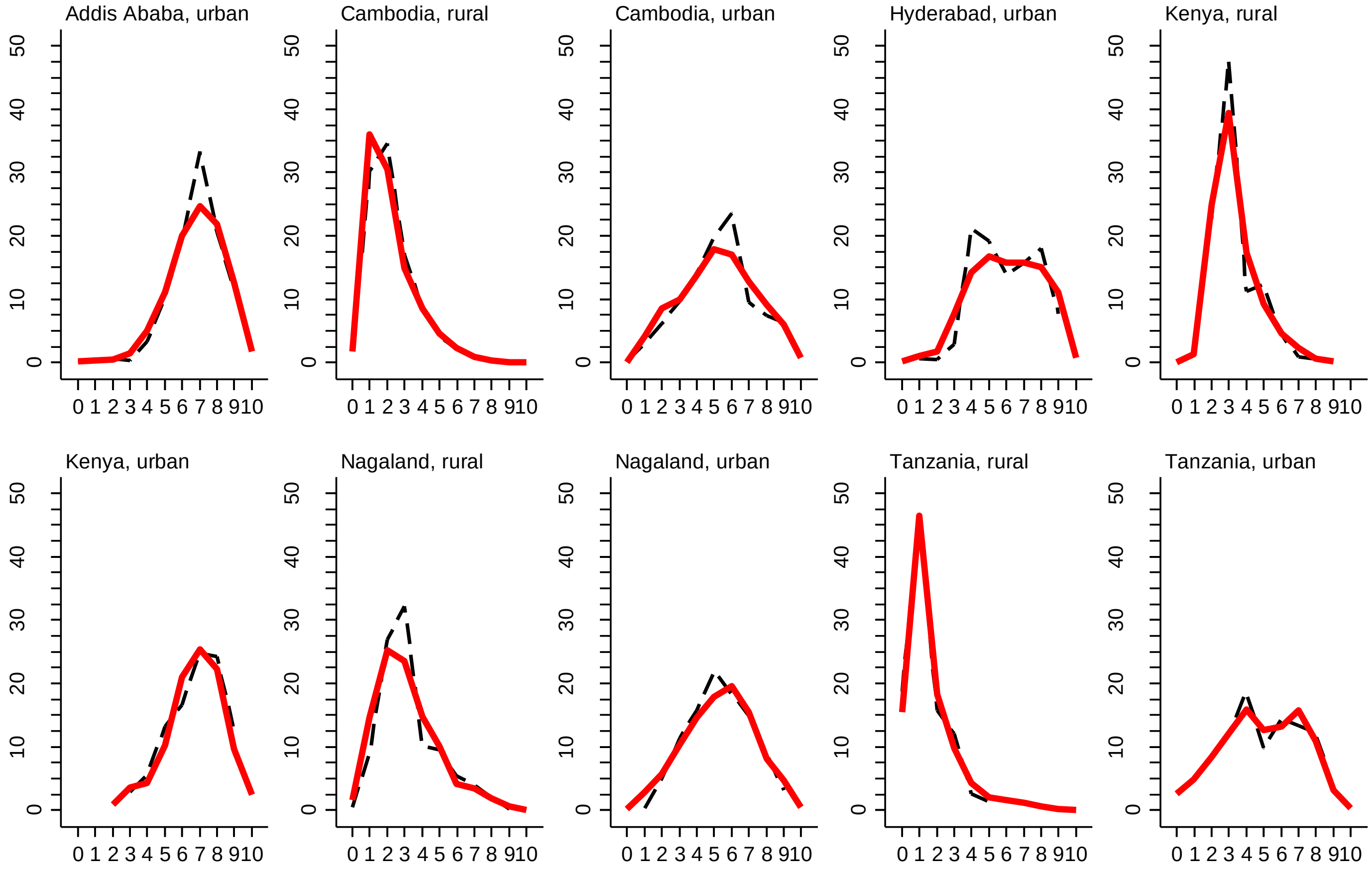

Distribution of actual (solid) and predicted (dashed) DHS wealth index scores

Note: Wealth index scores in each setting were re-scaled to range from 0-10.
